# One hundred years of rent control in Argentina: much ado about nothing

**DOI:** 10.1007/s10901-022-09932-6

**Published:** 2022-02-08

**Authors:** Alejandro D. Jacobo, Konstantin A. Kholodilin

**Affiliations:** 1 IEF FCE, Universidad Nacional de Córdoba, Blvd. Enrique Barros s/n, 5000 Córdoba, Argentina; 2 CICE (CIECS-CONICET), Av. Valparaíso s/n, 5000 Córdoba, Argentina; 3 DIW Berlin, Mohrenstraße 58, 10117 Berlin, Germany; 4 NRU HSE, Kantemirovskaya ul. 3, Saint Petersburg, 194100 Russia

**Keywords:** Argentina, Housing rents, Rent control, Rental market regulations, C21, E31, R38

## Abstract

Following World War I, rent control became a standard policy response to the housing shortage and the resulting rent increases. Typically, economists blame it for creating inefficiencies in the housing market and beyond. We investigate whether rental market regulations (including rent control, protection of tenants from eviction, and housing rationing) had any effects in a middle-income Latin American economy, such as Argentina. To answer this question, we take advantage of a wide range of housing market indicators and restrictive rental regulation indices covering almost one century. Using a standard OLS model and MARS, a nonlinear estimation technique, we find that rental market regulations have exerted a statistically significant negative impact on the growth rates of the real housing rents. However, they were only effective for short periods following both World Wars, when regulations were novel and particularly strong.

*Hace diez años, señora,*

*que soy aquí su inquilino*

*y nunca tanto tuvimos*

*estas broncas como ahora.*

*Usted protesta, señora,*

*con sus gritos imprudentes*

*que entera a toda la gente,*

*que quiere mi desalojo,*

*porque dice con enojo*

*que soy un tipo indecente..*.

From the milonga “El Inquilino”

## Introduction

Following World War I, rent control became a standard policy response to housing shortages and the resulting rent increases. Virtually all countries used such policies throughout twentieth century. A new wave of rent control came in 2020 as a response to the worldwide COVID-19 crisis (Kholodilin, 2020b). In Argentina, housing rents were frozen in March 2020. Typically, economists stigmatize it for creating inefficiencies in the housing market and beyond (Jenkins, 2009). In this study, we investigate whether it had any effects in Argentina, a middle-income Latin American country. To answer this question, we take advantage of a wide range of housing market indicators and restrictive rental regulation indices for Argentina covering more than 100 years. We find that rent control exerted a statistically significant impact on the performance of its housing market.

Sometimes referred to as a configuration of services that satisfy basic human needs, such as shelter, privacy, and identity, among other, housing has always been conceived as a need to be covered (Astorga González, 1995). Many households worldwide strive to be homeowners, since housing is related to the opportunity of having a protective element (against contingencies that affect their income, such as loss of employment or by family extensions). Housing usually means the availability of a capital that reduces uncertainties and it is occasionally a source of income (to rent a room or to set up an economic activity). Some authors even argue that in post-war years — mainly in Western Europe and Latin America and with nuances in both cases — the same state has been the promoter of a “culture of home ownership” by encouraging that citizens reside in their houses as owners rather than tenants (Pareja-Eastaway and Sánchez-Martínez, 2011, p. 53). Still there is a large part of population that lives in rental housing and spends a substantial portion of its income for rent. These people have typically lower incomes than the homeowners.

Since the turn of the twentieth century, the phenomenon of urbanization in Latin America, in general, and Argentina, in particular, is perceived as rapidly increasing, given the high rates of population growth and rise of the industrial activity, which was almost non-existent before the First World War and notably expanded in, at that time, a purely agricultural economy. Since then, Argentina has experienced a continual housing shortage.

Its constant population growth and the migration of its farmers to urban areas, coupled with an inflow of immigrants to the major cities of Argentina, are all elements leading to ever increasing demand for housing with respect to its supply. This inevitably led to increases in rental prices that inflicted painful financial blows to the tenant households.

Since 1921, the Argentinian government has reacted to these developments by introducing rent control policies, much like governments in many other countries. As a pendulum, phases of stronger regulations have been followed by those of the deregulation. On June 11, 2020, the Chamber of Senators of the Nation sanctioned under the number 27,551 the Ley de Alquileres, which is aimed at strengthening rental market regulations.^1^ Among other things, it provides for capping rent increases by an average growth rate of the consumer price index and the wage index as well as extending the minimum contract duration from two to three years. Thus, Argentina faced a new phase of increasing intensity of rental market regulations. However, this new measure appears to have strong negative byproducts. For instance, in the wake to these regulations 45% of landlords decided to stop letting their rental dwellings and to sell them. This led the Argentina’s parliament to discuss the possibility of phasing out the law for 180 days.^2^

While a simple outlook indicates that the whole world is dominated by the homeowners and, on average, in 81 countries from different regions about 70% households are homeowners, in Latin America homeownership growth rate has reached its peak in the 1990s and 2000, and it has started to decline. Concomitantly, the number of tenants has increased, and Argentina is along this line.

**Fig. 1:**
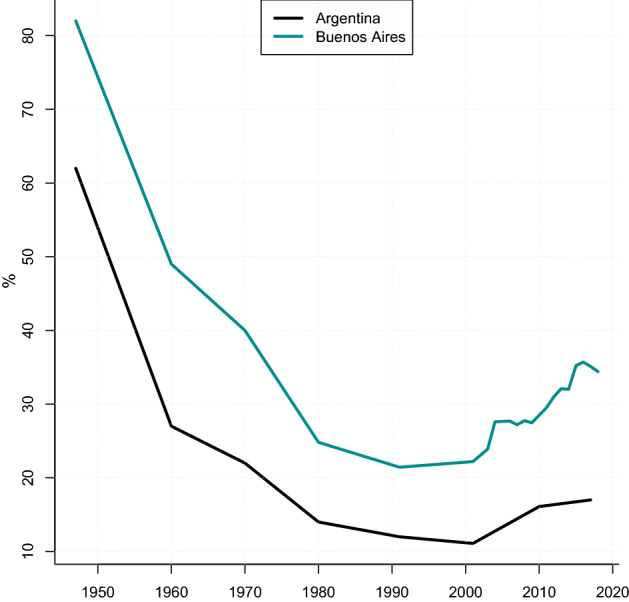
Tenant rate in Argentina, 1947–2018. *Note*: The tenant rate is measured as a percentage share of tenant households in the total number of households. *Source*: Benítez (2016), Cosacov (2012), Reese et al. (2014). (Color figure online)

Although Argentina seems to be a country of homeowners, the housing rental market is becoming more and more important, as seen in Fig.1. The share of tenant households used to be very high (62%) in 1947, then it declined until it reached its historic minimum in 2001 (about 11%). Since 2001, tenant occupancy rates have steadily increased, attaining 17% in 2017. Similar evolution is observable in Buenos Aires, its largest city, where the rate went from 82% in 1947 to 21% in 1991 and back above 34% in 2018.

According to Blanco et al. (2014), the number of tenants in Latin America has increased due to three reasons. First, the current housing policies based on subsidies are unsustainable from a fiscal perspective, insufficient to satisfy the demand, and inefficient in terms of the land-use patterns. Second, the growth of the cities has increased the scarcity of well-located urbanized land, which makes housing even less affordable. Third, the demographic changes (decrease in average size of households, rising divorce rates, and growth of the number of single-person households), coupled with a higher labor mobility associated with more flexible and globalized economies, make several households choose not to acquire their own home.

As to Argentina, additional reasons have stopped this trend and induced rental tenure. On the one hand, the increase in the price of houses (whose values are regularly estimated in US dollars) observed in recent decades was higher than the evolution of wages (also measured in US dollars). Indeed, at the beginning of the twenty-first century, real estate market has operated as a storage of value and the real-estate construction boom (channeling the surplus of agricultural income) produced in Buenos Aires, Córdoba, Rosario, and Santa Fe, etc., thus, stimulating house price increases (Carné, 2021).

On the other hand, the restrictions on mortgage credit have also played a role. Few households can meet income requirements from financial institutions in order to qualify for the credit.

In addition, the requirements imposed on the potential tenant by the landlord are often difficult to satisfy. Typically, the tenant must provide two guarantors (homeowners), who would be eager to pay his rent in case he will default. Moreover, the tenant must present his salary receipts that prove that his formal income is high enough. On top of this comes the provision of a safety deposit (refundable at the end of the contract) and the payment of a fee to the real estate agent.

In Argentina, interventions in the housing market use rent control and other restrictive measures were only effective during two short periods, being rather ineffective the rest of the time. For example, during Perón’s first presidency, the real estate market was virtually paralyzed. This was a consequence of a 1943 law that froze rents and interrupted evictions. These norms, in force until the fall of Perón in 1955, produced a severe contraction in the supply of dwellings for rent, because the profit expectations of real-state investors abruptly declined. Moreover, when Perón took the reins for his second presidential term, the economic outlook was very poor.

Under the circumstances, where rents were frozen and evictions not permitted, the real amount paid by occupants shrank to almost nothing and the market for houses for rental purposes almost disappeared. In what remained from the formerly large rental market, various means of avoiding the regulations were invented. For example, in order to get a place to stay, tenants agreed with landlords to pay the property taxes in addition to the rent. The fiscal appetite of the different governments also did not contribute to the effectiveness of rent controls. In fact, as landlords must pay income tax on rents received, contracted rents do not reflect reality: tenants pay the sum listed in the contract plus an additional amount through previously agreed promissory notes. Additionally, rental contracts require stamp duties. In almost all cases, the contract is not stamped by the parties in order to avoid this tax or is marked by half of the amount as agreed upon between the landlord and the tenant. The lack of stamp duties payment does not make a contract null. In case of eviction, the only risk that the landlord is bearing is the necessity to pay the stamp duties plus a penalty fee, which turns out to be ridiculously small when inflation is high.

There is an extensive literature on the effects of rent control, as shown in Table 1. Most studies address the US experience: nine out of 14 listed in the table. The rest focus on European countries, mostly Scandinavian (Denmark and Sweden). The majority of studies (nine) work with microdata, typically at the private household level. The studies concentrate on topics like residential mobility, misallocation of housing, rents, and homelessness. Few consider the effects on residential construction (Sims, 2007). The statistical methodology covers a wide range of techniques varying from simple cross-sectional regressions through panel data models and nonlinear models such as proportional hazard model, duration model, and logit. To our knowledge, there are no studies using econometric tools to analyze the effects of governmental regulations on the Argentinian housing market.

The contribution of the paper is threefold. First, it concentrates on the impact of rent control in a middle-income Latin American country. Second, this is the first assessment of rental regulations for Argentina and the overall region. Third, it uses a novel database with long-term time series of the Argentinian housing market.

This paper is organized as follows. Section 2 describes the data, their sources and transformations. Section 3 presents the estimation methodology. Section 4 reports and discusses the estimation results. Finally, Sect.5 concludes.

## Data

In this section, the sources, transformations and characteristics of data are described. The list of data with their sources are reported in Table 2. Below, we describe the data used in this study in more detail.

It is difficult to obtain overlapping time series for the variables under different base periods in Latin American countries over the long-run and Argentina is not an exception. It is typical that, once the base period is changed, the old time series (based on the previous base period) are discontinued and the new ones are not extended backward for a significant number of years. Frequently, a change in the base period usually reflects improvements in statistical procedures that began in Argentina during the 1960s when its statistical system started developing. This makes unclear whether the observed differences across base periods effectively reflect changes in the series or merely shows the peculiarities of statistical procedures. Regardless, we carefully describe the variables as shown in Table 2 and adopt the “second-best methodology” consisting in the simple “chaining” of the series as the only alternative available.

### Dependent variable

As our dependent variable, we take the average monthly rent index of a room in the City of Buenos Aires for the period 1914–1934 published by the *Departamento del Trabajo* of the *Ministerio del Interior* through its statistics division. From 1934 to 1961, the numbers also come from a contemporary index considering the consumption of a $4\times 4.5$ meters room by an unskilled worker’s family type (parents and two children under 14 years old) living in the City of Buenos Aires. We take the data from publications of the former *Dirección Nacional de Estadística y Censos* (DNEC, hereafter). This office also provides information for the 1961–1976 period and the variable turns out to be the rent of a house (excluding electricity) according to a survey on the living conditions of a working family (“familia obrera”) carried out in the capital of Argentina.

According to the official statistics that we follow, rent for 1977–1988 comes from the consumer price index and include housing expenses. The set of goods and services selected in the expenses are sanitary repairs, tiles, cement, bricks, wood, and paint. This group excludes other things (gas, the cost to refill a balloon of gas, kerosene, charcoal and electricity). The index considers the capital of Argentina. However, from March 1977 the index includes the capital of the country and 19 suburban communities. The information for the index comes from the *Encuesta Permanente de Hogares* (Permanent Household Survey) and the data are elaborated and published by the *Instituto Nacional de Estadística y Censos* (INDEC is the Spanish acronym for the National Institute for Statistic and Censuses). As for 1988–1999, the index measures the evolution of the monthly effectively rent paid by households, with expenses considered separately.

For 1999–2013, the survey of the rental prices is monthly and based on the division of the geographical area into work zones composed of the City of Buenos Aires and the Greater Buenos Aires.

Finally, for 2014–2017, data come from the *Dirección General de Estadística y Censos of the City of Buenos Aires*. The IPCBA (this is a Spanish acronym for Consumer Price Index of the City of Buenos Aires) contains the rent variable.

**Fig. 2:**
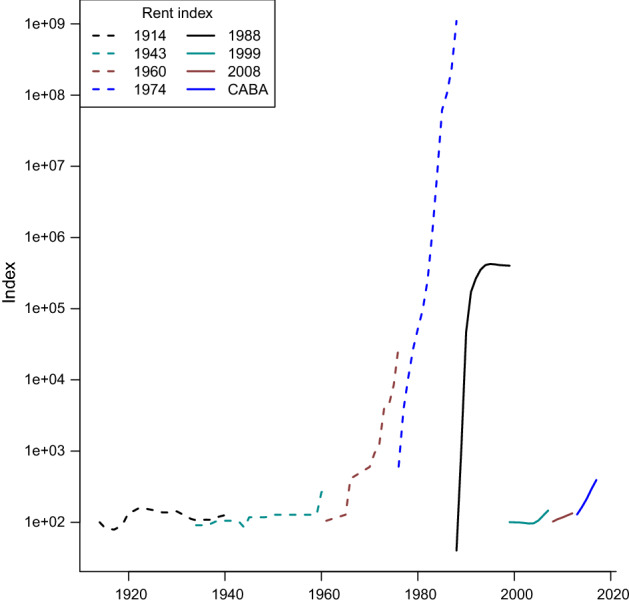
Various housing rental price indices, 1914–2017. *Note*: The numbers in the legend denote the basis years of the corresponding indices. CABA stands for Ciudad Autónoma de Buenos Aires. The vertical axis uses a logarithmic scale in order to make the low index values visible. (Color figure online)

**Fig. 3:**
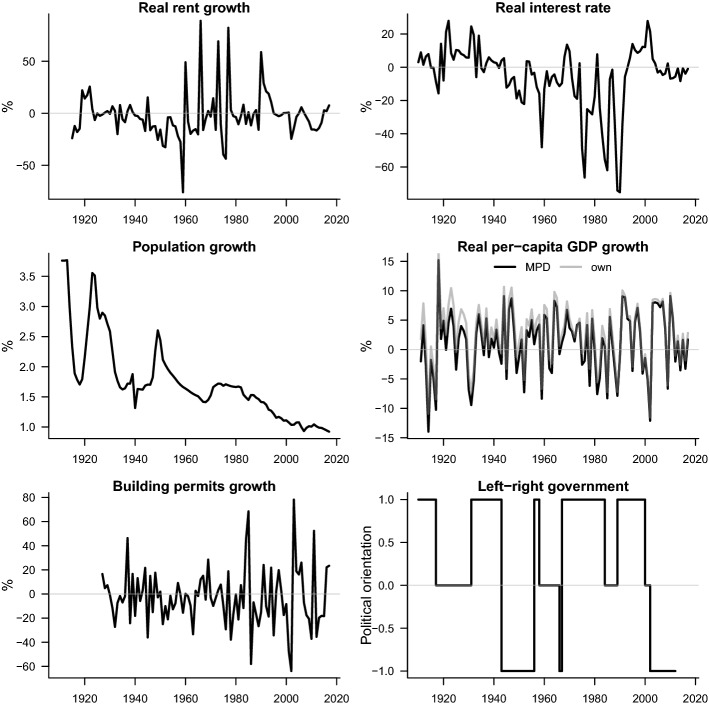
Dependent and control variables, 1910–2017. *Note*: The growth rates are computed as the percentage annual changes. The real values are obtained by dividing the nominal values by the consumer price index

The time series of monthly rent coming from different sources are linked to obtain a series covering the period 1914–2017, which is the first attempt of this sort for Argentina. The individual rental price indices with different basis years and coverage are shown in Figure 2. This nominal rent is deflated using the consumer price index. The growth rates of the resulting time series are displayed in Figure 3.

In order to account for the methodological differences across these seven periods, in all regressions below, six dummies are introduced, for 1914–1934, 1935–1960, 1961–1976, 1977–1988, 1989–1998, and 1999–2013, denoted as $D\_{\text{meth}}1, \ldots ,D\_{\text{meth}}6$.

### Control variables

*Interest rate*. From 1914 to 2008, the series represents the interest rate for 30-days loans in domestic currency (peso) to first-line companies (prime rate). From 2009 on, it is the 30-days discount rate to promissory notes. Ferreres et al. (2005) provides information for the 1910–2004 period. The series is updated with information from the web page of the *Banco Central de la República Argentina*.

*Gross Domestic Product* As usual, the series is the sum of good and services produced by the Argentine economy during a year. Ferreres et al. (2005) covers 1910–2004, while the national accounts compiled from INDEC allow us to properly update the series.

*Consumer Price Index* The series is from Ferreres et al. (2005), who presents values up to 2004. However, to continue the series, we have to consider the government’s intervention in the Argentine Statistics Bureau (INDEC) from 2007 through 2015. During these years, the government started reporting official statistics that were systematically below the unofficial ones. We follow Cavallo and Bertolotto (2016) to update the annual series.

*Population* This variable indicates the projected population in thousands of persons. From 1910 to 2004 the data come from Ferreres et al. (2005), while the series up to 2017 are from *Dirección General de Estadísticas y Censos de la Ciudad de Buenos Aires*.

*Building permits* This variable broadly corresponds to the number of building permits, i.e., the administrative procedures through which the authorization for the construction of a building is requested. Each building permit generally corresponds to a work, so this variable largely reflects the number of buildings authorized. The source is the *Revista Económica* from the Banco de la Nación Argentina for the 1926–1934 period. A special request by the authors was made to the Dirección General de Estadística y Censos of the City of Buenos Aires for 1934–1943 data. From 1944 to now, data proceed from the building series of the national statistical office of Argentina through its different names (Dirección, Nacional del Servicio Estadístico, Dirección Nacional de Investigación Estadística y Censos, Dirección Nacional de Estadística y Censos, and INDEC). Unfortunately, to our knowledge, information about building permits is not available prior to 1926.

*Demographics* We use two demographic variables: the population growth and the growth of the number of marriages. This represents the demand side of the housing market. A large population represents a higher demand for the living space. Likewise, the number of marriages proxies the formation of the new households, each of which, at least in theory, requires a separate dwelling. It is expected that these variables should exert a positive impact on the housing rents.

*Political orientation of the government* The researchers both in political science and economics have used the left-right spectrum to explain political decision making, starting with Downs (1957). It can be expected that the leftist governments are more inclined to expand social policies, including rent control. The proxy used here is the orientation of the political party of the head of the government belongs. This variable is constructed by Brambor et al. (2017). It takes three values: $-1$ for left, 0 for center, and 1 for right. Thus, the expected sign of the regressor is negative.

The evolution of control variables between 1910 and 2017 is shown in Fig.3. The real rent growth is quite volatile with several large peaks between 1960 and 1980. The real interest also varies wildly between − 70 and 20. It is very negative between 1960 and 1990 due to a large hyperinflation in that period. The population growth shows a secular decline with several cycles. Between 1910 and 2016, it dropped from 3.5 to 1% a year. The two indicators of the real GDP (Maddison Project Database and the indicator compiled by authors from different sources) have very similar dynamics, the compiled indicator showing slightly higher growth rates than that from the MPD. The building permits growth is also quite volatile, with the variation increasing toward the end of the sample. It is also a variable that starts in 1927, much later than all other time series. Finally, the left-right government index of Brambor et al. (2017) shows the fluctuations in the political orientation of the heads of government in Argentina. Most governments appear to belong to the right wing of the political spectrum. Only three times the leftists managed to gain control over the government: 1943–1955, 1966, and 2002–2012.

### Regulation indices

**Fig. 4:**
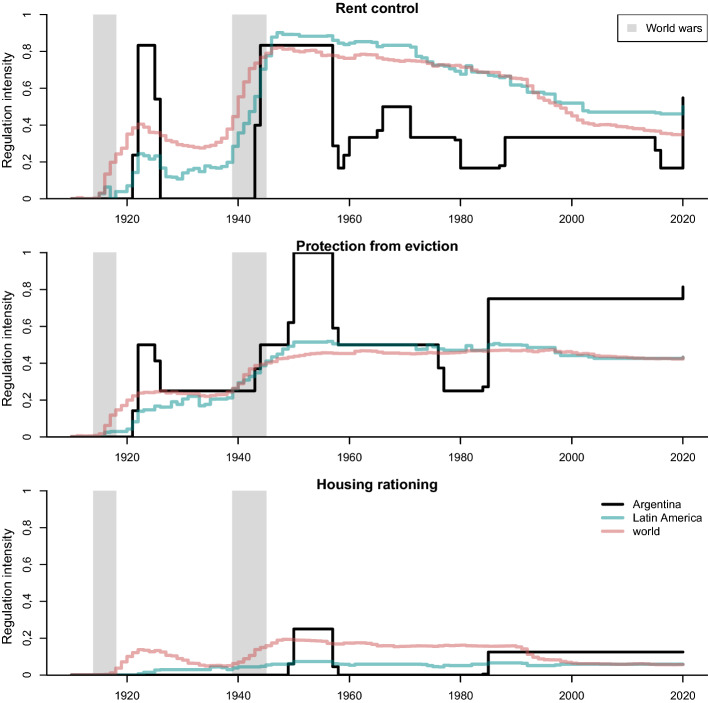
Rental market regulation indices in Argentina, 1910–2020. *Note*: The lines reflect the intensity of the restrictive rental housing market regulations (rent control, protection of tenants from eviction, and housing rationing) in Argentina (black line), Latin America and Caribbean (green line), and the world as approximated by the 133 countries and states (red line). The higher the index value the more intense the governmental intervention in the rental housing market. Two gray bars correspond to both world wars. (Color figure online)

This study focuses on the effects of governmental policies. Therefore, we need measures of their intensity. For this purpose, we use the restrictive rental market regulations indices elaborated by Kholodilin (2020b) and Weber (2017). These indices cover three types of regulations: rent control, tenure security, and housing rationing. All three indices vary between 0 and 1: the higher the index, the more intense the regulation. The indices are constructed for Argentina based on a thorough analysis of the corresponding legal acts. Table 4 summarizes all relevant laws underlying the rental market regulation indices utilized in this study. Figure 4 depicts the evolution of the three indices between 1910 and 2017, with shaded areas denoting both World Wars. For comparison purposes, it also shows the evolution of the indices for Latin America and the world.

Rent control index measures the intensity of restrictions imposed on the level of rent and its rate of increase. The index is computed as a simple average of six binary variables: 1) rent level control, if rents are set by some governmental body, court, arbitration council or similar and not by a free negotiation between tenants and landlords; 2) nominal rent freeze, if rent increases are prohibited; 3) real rent freeze, if rent increases are allowed but cannot exceed the growth of a cost of living index; 4) intertenancy decontrol, if a tenant change implies a deregulation of the formerly regulated dwelling; 5) other specific rent decontrol, if rent control is not applicable to certain types of dwellings (e.g., newly built or luxury ones); and 6) specific rent recontrol, if stricter rent control is applied to types specific households (e.g., low-income or having military as their member), areas (e.g., communities with tight housing market), or types of landlords (e.g., big ones). These binary indices take the value 1, if in particular year a corresponding restriction exists or exception is absent. Using the simple average implies equal weights of all the binary indices and, thus, their equal impact on the resulting composite index. Using different weights would be arbitrary, since it would be difficult to justify why certain binary indices should obtain higher weights. Moreover, even if some objective weighting rule could be found, it would be difficult to implement it due to the lack of data, when it is done on an international scale. In addition, the economists distinguish between first- and second-generation rent controls (Arnott, 1995). The first generation implies a rent freeze, when rents are fixed at some level. For instances, in Argentina rents were frozen three times: 1921–1924, 1943–1956, and 1965–1970, at the January 1, 1920 level, at the December 31, 1942 level, and at the previous contract level, respectively. Under the second-generation rent control, the rent level, as a rule, is not frozen; instead, the restrictions are imposed on the growth rate of rent, which is typically anchored to some measure reflecting the cost of living. In this way, lawmakers guarantee that the real rental revenues of the landlords are not eroded by inflation. In Argentina, in 1970, rent increases were capped by the rate of increase of the official index of living costs (*índice de costo de vida*). However, between 1987 and 2014, the rents were nominally frozen, for the government did not allow rent to be indexed by inflation in order to avoid an inflationary spiral. In terms of the index construction, the first-generation rent control implies that both rent control level and nominal and real rent freezes exist, while under the second-generation rent control, only real rent freeze exists.

The tenure security index reflects the degree of protection that tenants have from evictions by landlords. The main instruments of protection are 1) eviction protection during term or period; 2) eviction protection at the end of term or period; 3) imposition of a minimum duration of rental contracts; and 4) prohibition of short-term (less than one year) tenancies. Between 1921 and 1949, the first two tools were applied in Argentina: contracts could be automatically prolonged by tenants and landlords could only evict them, if they had justifiable reasons to do so. These reasons included: 1) non-payment of rent; 2) abusive use of the rented premises; 3) tenants initiating scandals (*escándalo*); 4) the owner needs the dwelling for himself and his family; or 5) the owner plans to rebuild the house, having low housing capacity, in order to create more dwellings, etc. In 1949–1957, the restriction on the minimum duration of rental contracts was added. In 1957, this requirement was abandoned. Finally, from 1976 on, the automatic prolongation of existing rental contracts was no longer provided to the tenants. However, during the term of those contracts, the tenants are still protected from eviction.

The housing rationing index measures the intensity of redistribution of the existing housing stock. In Argentina, between 1949 and 1965, three such policies were applied: 1) obligatory registration of vacant dwellings by landlords and subletting tenants within 15 days; 2) landlords are required to let their dwellings within 30 days; and 3) in the Federal Capital and National Territories, the authorities can requisition vacant dwellings.

The rental housing market regulation indices have their weaknesses, being a result of a tradeoff between the feasibility and the complexity of the real world. First, they are based on the formal laws and do not take into account their enforceability related to the effectiveness of the legal system and the degree of the legal literacy of the society. Second, these indices account for a limited number of relevant characteristics, skipping some other features. For example, they do not include the regulations concerning security deposits or subletting. Third, rental regulations typically apply to a specific segment of the housing market. If the size of this segments changes over time (for example, the rental market squeezes), the application sphere of regulations changes, too. However, due to the data limitations it is not always possible to take this into account.

All variables are tested for stationarity. The results of the augmented Dickey-Fuller unit-root tests are reported in Table 5. For the real interest rate (RIRate), the null hypothesis of unit-root (presence of random walk) can, in most cases, be rejected at conventional significance levels. Other variables become stationary after taking first differences. Only the growth rate of population (DLPop) appears to be non-stationary reflecting a secular decline in the speed of expansion of Argentina’s population. In addition, we tested our series for cointegration using the Engle-Granger test (Engle and Granger, 1987). The alternative hypothesis of the existence of a cointegrating relationship between the levels of variables could not be confirmed at 10% of significance. Therefore, we are going to use the growth rates in our regressions.

## Econometric methodology

In order to investigate the potential impacts of rental regulations on the real growth rates of housing rents, we use different estimation approaches.

First, we take advantage of a simple ordinary least squares (OLS) model:

1 $$\begin{aligned} y_t = \alpha + \rho y_{t-1}+ \sum _{k=1}^K\beta _k x^C_{k,t-1} +\sum _{l=1}^L \gamma _l x^R_{l,t-1} + \varepsilon _t \end{aligned}$$

where $y_t$ is the growth rate of real rent in period *t*; $x^C_{kt}$ is a *k*-th control variable; $x^R_{lt}$ is an *l*-th regulation index (our focus variable); $\varepsilon _t$ is the disturbance term; and $\alpha$, $\beta$’s, $\gamma$’s, and $\rho$ are parameters to be estimated. Note that explanatory indices are taken with a lag in order to avoid possible endogeneity. Here, we use a dynamic model in order to account for the possibility of persistent rent growth. The advantages of the linear model are its simplicity and easy interpretation. It is also the most widely used model in the literature. A big disadvantage is that linear models ignore possible nonlinear effects and sometimes do not find any effects at all, if the relationship between dependent and explanatory variables changes over time. This can be especially true, when the policy effects are investigated, since both policies and society undergo changes. Moreover, the society can adapt to the policies and, thus, render them less effective, as the famous Lucas critique suggests (Lucas, 1983).

Second, we employ a multivariate adaptive regression splines (MARS) algorithm. This is a nonparametric piecewise regression technique that was introduced by Friedman (1991). It is especially useful for identifying nonlinearities in regression models. These are modeled using potentially different slopes for each predictor. Thus, unlike the linear regression, MARS does not assume that coefficients are stable across the entire range of each variable and instead uses splines in order to fit piecewise linear continuous functions. This is very useful when considering long-run economic processes, where policy responses may be subject to structural breaks. The main advantages of MARS compared to other nonlinear models (e.g., polynomial models) are the simplicity of the resulting econometric model, its interpretability, and automatic model selection.

Here, we use matrix notation in order to formulate MARS. The dependent variable $Y=(y_1,y_2\ldots , y_T)'$ is regressed upon a set of potential explanatory variables $X=(X_1,X_2,\ldots ,X_K)$ with $X_k=(x_{k1},x_{k2},\ldots ,x_{kT})'$, where $k=1,2,\ldots ,K$ is the number of regressors and $t=1,2,\ldots ,T$ is the time index. The MARS uses the so-called *basic functions* (BF) of the form $(x-c)_{+}=\max \{0, x-c \}$ and $(c-x)_{+}= \max \{0,c - x \}$, where the subscript “+” means that the function takes only a positive value or zero in case of negative difference. In other words, the threshold *c* splits the corresponding explanatory variable in two subsets: the values above and below *c*. Thus, two new variables emerge: 1) values exceeding *c* or zeros and 2) values below *c* or zeros. The sum of these two variables is equal to the original explanatory variable. When included into regression instead of the original variable, this pair of newly constructed variables allows modeling the impact of the explanatory variable in a nonlinear way — as two linear pieces with different slopes. Such pairs of linear functions are called “hinge functions” and the constant *c* denotes a knot, where the slope changes. The collection of all possible BFs, ${\mathcal {C}}$, is used to construct the following model:

2 $$\begin{aligned} {\mathcal {C}}=\{(x-c)_{+}, (c-x)_{+}\} \ \ \text {with} \ \ c \in \{ x_{k1}, x_{k2},\ldots , x_{kT}\} \; \ \text {and} \ k=1,2,\ldots ,K \end{aligned}$$

Each function is piecewise linear with a knot *c* at every $x_{kt}$, and, in case if all input values are distinct, there are *TK* hinge functions, or equivalently 2*TK* basic functions. The model-building strategy is similar to a classical forward stepwise regression using as inputs the functions from the set ${\mathcal {C}}$ and their products. The complete MARS model is formulated as:

3 $$\begin{aligned} Y = \beta _0 + \sum _{k=1}^{K} \beta _k h_k(X) + \epsilon \end{aligned}$$

where $h_k(X)$ is either a BF; or a product of two or more such functions, if interactions between variables are permitted; or the original explanatory variable, if it exerts a linear impact on the dependent variable; and $\epsilon$ is an error term. Here, however, we do not consider interactions. The coefficients $\beta _k$ are estimated by minimizing the sum of squared (residual) errors (SSE), similar to a standard linear regression model.

The model-training process iteratively selects and adds some hinge functions to the model (or the original explanatory variable). During each step of the training process, MARS selects new terms that minimize the SSE using OLS. In this forward pass, the MARS algorithm starts with a single model including only the intercept term $\beta _0$. At each subsequent step, a pair of hinge functions and an original explanatory variable are selected and added to the model. The forward pass continues until it meets one of many conditions such as: 1) the maximum number of model terms (chosen by the user) before pruning is reached; or 2) when adding a term $R^2$ changes by less than a threshold value selected by the user (e.g., 0.001). In general, at the end of this process, we have a large model in the form described by equation (2). The MARS model obtained in this forward pass is adaptive and can exhibit a great degree of flexibility that can ultimately result in overfitting, if no measures are taken to counteract it. To solve the overfitting problem and build a model with better generalization ability, a pruning procedure must be applied. The pruning implies that a one-at-a-time backward deletion procedure is applied during which basis functions with the least contribution to the model are repeatedly eliminated.

## Results

The OLS regression estimation results are shown in Table 6. Columns (1) and (2) report the results of two models estimated using OLS. Both models include a subset of control variables that are typically included in the regressions explaining the dynamics of housing rents regardless of whether their coefficients are statistically significant or not. In addition, model (1) contains the Rental Market Regulation Index (RMRI) and housing rationing index, while model (2) contains rent laws, tenure security, and housing rationing indices. Given that RMRI is a simple average of rent laws and tenure security indices, we can only use it together with the housing rationing index in order to avoid multicollinearity. Both the studentized Breusch-Pagan test and non-constant variance score test have very low *p*-values pointing out to the existence of heteroskedasticity.^3^ The Durbin-Watson test could not reject the null of no autocorrelation,^4^ which is not a big surprise, given that we use dynamic models. Finally, the Shapiro-Wilk test of normality rejects the null hypothesis of the normal distribution of the regression residuals.^5^ Therefore, we employ bootstrap approach to calculate consistent standard errors. The bootstrapping is done using 300 replications.^6^ The autoregressive term is only significant in model (2). However, it has a negative sign, indicating rather erratic fluctuations of real rent growth rates instead of persistent dynamics. The only control variable that is statistically significant is the real interest rate. None of the regulation indices are statistically significant. This implies that rental housing market regulations have not exerted any linear effect on rents in Argentina. However, it is possible that due to the differences in the rent control regimes between different periods, there might be effects in some periods and opposite or no effects in other periods, cancelling each other over the whole period. In order to verify this possibility, we take advantage of a nonlinear model.

**Fig. 5:**
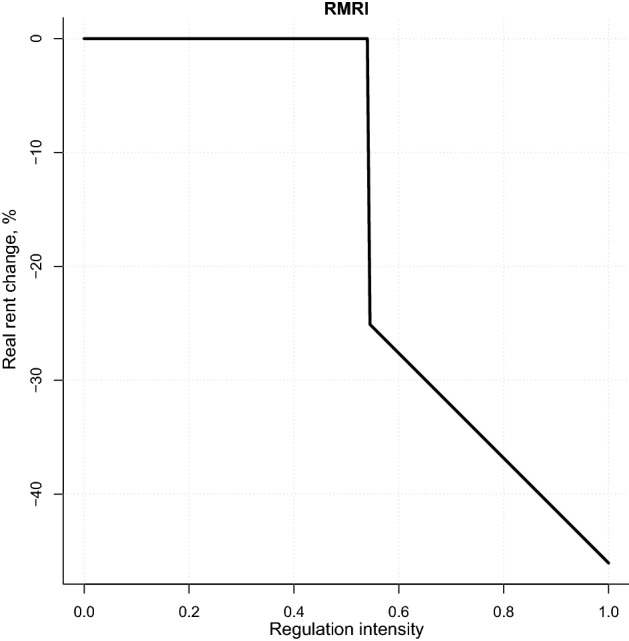
Regulation effects on real rent growth. *Note*: The figure depicts the estimated effects of rental market regulations on the real rent growth. The intensity displayed on the horizontal axis varies from 0 (no regulations) to 1 (very strict regulations)

Columns (3) and (4) report the same two models estimated using MARS. The nonlinearity is only allowed for regulation indices. The automatic selection algorithm dropped autoregressive term from both nonlinear models. The models always include the real interest rate, which appears to exert a statistically significant negative impact on the growth of real rents. In model (3), in addition, the Rental Market Regulation Index is contained. It has a negative effect on real rents, when it exceeds 0.542 and no effect below this threshold, see Figure 5. Such regulation intensity is only achieved during two episodes: 1923–1925 and 1945–1957. This is the aftermath of both World Wars.

The first period is consistent with the introduction of rent control; an innovation that probably tended to act as a surprise for the economy at that time and consequently exercised its effects. The second period coincides with the presidency of Perón when Argentina experienced a turbulent subordination of the economy to politics (a “hyper-politization of economic life”, in terms of (Gerchunoff and Díaz Alejandro, 1989, p.59). This subordinate position was one of the reasons for the economic enlargement of the public sector and the upsurge of controls, which clearly included rents. The aim and priority of the Perón’s government were to rapidly modify income distribution to create an economic order capable of preserving the distributive model pursued. Thus, rental regulations seem to be effective only when they are a novelty or a very strong policy, as they were during these periods.

The results presented here are robust to different specifications. First, the linear models reported in Table 7 use different lags of the control variables and regulation indices: between 2 and 4 years. The idea is that the market participants do not react immediately to the policy changes, but rather with a lag of several years. Moreover, these models include additional variables with respect to our basic model, such as the growth of consumer prices and left-right index. None of these additional variables turns out to be significant. Likewise, the regulation indices remain mostly statistically insignificant, except for model in column (4) that includes the third lags pointing to a possible transitory reaction three years after the regulation was enacted.

Second, Table 8 reports estimation results of linear models including some alternative proxies. The growth rate of per-capita real GDP is approximated alternately by the estimate of the Maddison Project Database and by the estimate that the authors compiled from various national sources. Moreover, instead of the population growth we use the growth of marriages. However, again, neither these variables nor regulation indices are statistically significant.

**Fig. 6:**
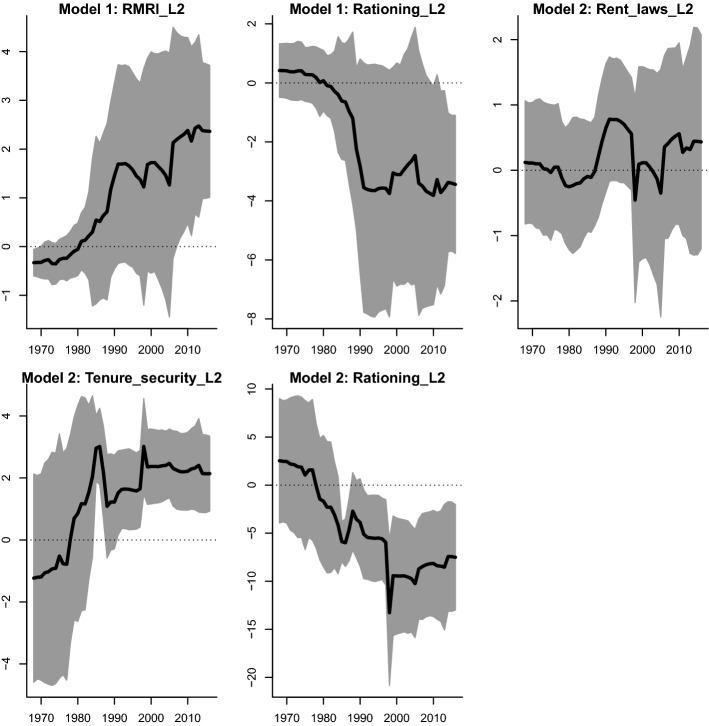
Coefficients of regulation indices estimated using rolling windows. *Note*: The regression coefficients displayed as a black line are estimated using a rolling 40-year window. The horizontal axis shows the last year of each 40-year period. For example, the estimate shown for 1980 was estimated using the period 1941–1980. The gray shaded areas show the 95%-confidence intervals, while dotted line corresponds to 0

Third, we estimated the model including regulation indices and and control variables, such as real interest rate, population growth, real GDP growth, building permits growth, as well as methodological-differences dummies using the 40-year rolling dummies. Our first subsample encompasses the period from 1929 to 1968, the second subsample covers 1930–1969, and so on. For each such subsample, both models (1) with RMRI and housing rationing; and 2) with rent laws, tenure security, and housing rationing) are estimated and the coeffcients at regulation indices are stored. At the end, we obtain a time series for each coefficient estimate together with its 95% confidence interval; see Fig.6. It can be seen that the coefficient of some regulation indices changed dramatically between the first (1929–1968) and the last subsample (1978–2017). For example, the coefficient of RMRI increased from $-0.33$ to 2.36. It was statistically significant at the very beginning and then als at the end of the sample. The tenure security index became positive and statistically significant from the 1980s. The housing rationing index declined strongly across the time and became negative and statistically significant in the last two decades of the sample. This confirms our hypothesis about the nonlinear effects of rental housing market regulations and explains why the linear model estimated over the whole sample produces statistically insignificant results.

## Conclusions

As most Latin American economies, since the turn of the twentiieth century Argentina has experienced a constant housing shortage. Its continuous population growth and the migration of its farmers to urban areas, together with an inflow of immigrants to the major cities, are all elements leading to ever increasing demand for housing with respect to its supply. The government has reacted to these developments by introducing rent control policies.

In this study, we analyze the effects of rental market regulations in Argentina on the growth of real housing rent. We use a novel database with long-term time series of the Argentinian housing market and take advantage of a wide range of indicators as well as restrictive rental regulation indices for Argentina covering more than 100 years.

We find that these regulations did exert a statistically significant impact on rent dynamics. They appear to dampen real rent increases. However, these effects are observed during two short periods following both World Wars: 1923–1925 and 1945–1957. This means that rental market regulations are effective when both rent control and tenure security are novel or very strong. However, we do not consider potential effects of rent control on other aspects of the housing market, such as residential construction, quality of housing, and homeownership rate.^7^ These effects can counteract the policy-driven declines in real rents making such interventions far from desirable as a tool of rendering the housing affordable.

Hence, the policy recommendation for the governments of middle-income Latin American countries would be to not rely on rent controls and too strong tenure security, since when they are strong, they generate various negative byproducts, while, when they are weak, they hardly slow down rent increases.

## Appendix

See Tables 1, 2, 3, 4, 5, 6, 7 and 8.

Figures 1, 2, 3, 4 , 5 and 6.

**Table 1:** Literature on rent control effects

Study	Place and period	Type of data	Method	Effects
Ault et al. (1994)	New York, 1968	Micro, housing vacancy survey	Cross-sectional regression	Lower mobility
Munch and Svarer (2002)	Denmark, 1992–1999	Micro, 10% random sample of adult population	Proportional hazard model	Lower mobility
Krol and Svorny (2005)	New Jersey, 1980, 1990, and 2000	Census tract data	Cross-sectional regression	Higher commute times
Bettendorf and Buyst (1997)	Belgium, 1920–1939	Macro	Rotterdam demand model	Redistribution of household expenditure toward non-housing consumption
Sims (2007)	Massachusetts, 1985–1998	Micro, housing survey		Little effect on new housing construction, shift units away from rental status, and lower rents
Glaeser and Luttmer (2003)	New York City, 1993	Micro, housing surveys	Cross-sectional regression	Misallocation of housing
Autor et al. (2014)	Cambridge (Massachusetts), 1995	Micro, parcels of land	Cross-sectional regression	Large and significant positive indirect effect of decontrol on the valuation of properties that were exposed to controlled units
Moon and Stotsky (1993)	New York, 1978–1987	Micro, housing units	Tobit and panel data model	Decline in the quality of rent-controlled dwellings or reduction of the chances that housing units improve in quality
Grimes and Chressanthis (1997)	200 US cities, 1990	Macro, census data	TSLS	Higher homelessness
Early and Olsen (1998)	44 US metropolitan areas, 1985–1988	Macro, housing survey and micro, homelessness survey	TSLS and logit	Net effect: lower homelessness
Olsen (1972)	New York, 1968	Micro, survey of housing units	Cross-sectional regression	Increase of cost of landlords is larger than increase of real income of households in controlled units, hence, negative net cost for society
Svarer et al. (2005)	Denmark, 1997–2000	Micro, 10% sample of adult population	Competing risks duration model	Individuals occupying controlled units are less (more) likely to accept jobs outside (in) their local market labor, hence, longer unemployment duration
Skak and Bloze (2013)	Denmark, 2004	Micro, 20% sample of the rental market	Hedonic regression	Significantly lower rents in the controlled sectors and to a negligible increase in the uncontrolled rent
Wilhelmsson et al. (2011)	Sweden, 1994–2004	Macro, municipalities	Panel data model	Lower vacancy rates
Diamond et al. (2019)	San Francisco, 1990–2016	Micro, address history of individuals	Difference-in-differences	Decreased mobility of tenants in controlled dwellings; conversion of rental dwellings to condos

**Table 2:** Data definitions and sources

Variable	Period	Description	Source
Rent	1914–1934	Average monthly rent of a room in the city of Buenos Aires	Ministerio del Interior (1949), Investigaciones Sociales
Rent	1934–1956	An unskilled worker’s family type (parents and two children under 14 years old) in the city of Buenos Aires consuming a 4$\times$4,5 $\hbox{m}^2$ room	Dirección Nacional de Estadística y Censos (1957), Índices del costo del nivel de vida, actividad industrial y costo de la construcción
Rent	1956–1961		Dirección Nacional de Estadística y Censos, Boletín Mensual de Estadística de la República Argentina, various issues
Rent	1961–1976	Considers the rent of a house, excluding electricity, according to the survey on living conditions of a working family (“familia obrera”)	Dirección Nacional de Estadística y Censos, Boletín Mensual de Estadística de la República Argentina and Boletín Estadístico Trimestral, various issues
Rent	1977–1984	Rent considers the expenses for housing. The set of goods and services selected in housing expenses are: rent, sand, sanitary fixtures, tiles, cement, bricks, wood, and paint. This group does not include fuel (gas, the cost to refill a balloon of gas, kerosene, charcoal) and electricity	Instituto Nacional de Estadística y Censos. Fascículo Índice de precios al consumidor y Salarios industriales, various issues
Rent	1985–1988		Instituto Nacional de Estadística y Censos, Estadística Mensual, various issues
Rent	1989–1995	Measures the evolution of the monthly effectively rent paid by households that live in rented homes. The rental service covers the accommodation, excluding the payment of services (electricity, gas, water, heating, use of the telephone). It does not include: repair and maintenance of the dwelling (materials and labor), electricity, sanitary services, gas and other fuels	Instituto Nacional de Estadística y Censos Estadística Mensual, various issues
Rent	1996–1999		Instituto Nacional de Estadística y Censos, Indec Informa, various issues
Rent	1999–2008	Rent, excluding fuels for housing, electricity, water and sanitary services, materials, as well as work for repairs and common expenses of the home	Instituto Nacional de Estadística y Censos. Indec Informa, various issues
Rent	2008–2013	Rental of housing excludes: basic services and fuel for housing (gas in carafe, natural gas by network, kerosene); electricity; water and sanitary services (sewers, and storm drains); materials for repairs; common expenses of housing (expense)	Instituto Nacional de Estadística y Censos. Indec Informa, various issue
Rent	2014–2017	Rental of the house, excluding materials for the repair of the house (pre-mixed plaster, faucet set, ceramic floor, and interior paint), services for the repair of the house (locksmith, gas electrician, painter, plumber), running water supply, common expenses for housing (expense), electricity and gas (packaging and network).	Dirección General de Estadística y Censos de la Ciudad de Buenos Aires: https://www.estadisticaciudad.gob.ar/eyc/?p=28446
Building permits	1926–1934	Number of administrative procedures through which the authorization for the construction of the building is requested. This variable largely reflects the amount of works that are authorized	Banco de la Nación Argentina (1934), Revista Económica
Building permits	1934–1943		Available upon request from Dirección General de Estadística y Censos de la Ciudad de Buenos Aires
Building permits	1943–2017		Dirección Nacional del Servicio Estadístico, Anuario estadístico de la República Argentina, Tomo I, Compendio 1949-1950; Dirección Nacional de Investigaciones, Estadística y Censos, Sintesis Estadística Mensual de la República Argentina, various issues; Dirección Nacional de Estadística y Censo, Boletín Mensual de Estadística de la República Argentina, various issues; Dirección Nacional de Estadística y Censos, Boletín de Estadística, various issues; Instituto Nacional de Estadística y Censos, Boletín Estadístico Trimestral, Edificación, Permisos para Construcciones Privadas and Indec Informa, various issues
Consumer price index	1910–2004	Measures the variations in prices in Argentina	Ferreres et al. (2005)
Consumer price index	2005–2017		Cavallo and Bertolotto (2016), https://dx.doi.org/10.2139/ssrn.2787276
Interest rate	1914–2004	From 1914 to 2008 is the rate for 30 days loans to first line companies. From 2009 onward, it is the interest rate of discounted promissory notes	Dos siglos de Economía Argentina
Interest rate	2005–2017		Banco Central de la República Argentina: www.bcra.gob.ar
GDP	1910–2004	Gross Domestic Product of Argentina	Ferreres et al. (2005)
GDP	2004–2017		Instituto Nacional de Estadística y Censos. Cuentas nacionales
GDP	1911–2017	Real GDP per capita in 2011 dollars	Maddison Project Database (MPD) 2020
Marriages	1910–2010	Registered marriages in the city of Buenos Aires (Capital Federal)	Dirección General de Estadísticas y Censos de la Ciudad de Buenos Aires, Las estadísticas históricas de la Ciudad de Buenos Aires 1810-2010
Marriages	2011–2013		Dirección General de Estadísticas y Censos de la Ciudad de Buenos Aires, Matrimonios en la Ciudad de Buenos Aires, Años 1990-2013. Informe de resultados 763
Marriages	2014–2017		Dirección General de Estadísticas y Censos de la Ciudad de Buenos Aires, Anuario Estadístico de la Ciudad de Buenos Aires, año 2017
Pop	1910-2017	Total population, 1000 persons	Instituto Nacional de Estadística y Censos
Left-right government	1910-2012	The orientation of the political party to which the head of government belongs; the variable takes three values: left, center, and right	Brambor et al. (2017)

**Table 3:** Descriptive statistics of variables used in regressions

Variable	Description	Period	Minimum	Mean	Maximum	Standard deviation
DLRRent	Growth of real rent	1915–2016	− 0.759	− 0.025	0.889	0.223
RIRate	Real interest rate	1910–2017	− 75.227	− 5.583	27.946	19.670
DLPop	Population growth	1911–2017	0.009	0.017	0.038	0.007
DLgdppc	Growth of real GDP per capita (MPD)	1911–2017	− 0.140	0.011	0.152	0.053
DLRGDP	Growth of real GDP per capita (own construction)	1911–2017	− 0.115	0.027	0.168	0.053
DLPermits	Growth of building permits	1927–2017	− 0.639	− 0.028	0.783	0.239
DLCPI	Growth of consumer prices	1911–2017	− 0.172	0.327	3.459	0.594
Left_right_BLS	Left-right government	1910–2012	− 1	0.233	1	0.819
Rent_laws	Rent laws index, [0,1]	1910–2016	0	0.303	0.833	0.269
Tenure_security	Tenure security index, [0,1]	1910–2016	0	0.493	1	0.279
Rationing	Housing rationing index, [0,1]	1910–2016	0	0.055	0.250	0.077

**Table 4:** Rental market legal acts

Date and law title	Application sphere	Rent control	Protection of tenants from eviction	Housing rationing
1921-09-15 / 1921-09-19 — Ley 11.156 Locación urbana. Modificación de los artículos 1504, 1507, 1509, 1583, 1604 y 1610	Objects: house (*casa*), room (*pieza*), or apartment (*departamento*) let or sublet with contract duration $\le$2 years.	Setting: rents are fixed for the period of contract prolongation. Subrent increase cannot exceed 20%.	Automatic prolongation by 1.5 years, for furnished houses and chambers the prolongation corresponds to the payment frequency. Non-prolongation reasons: 1) non-payment of rent during two consecutive periods; 2) dishonest or contradicting the good customs behavior of tenant; 3) use of dwelling for other than stipulated purposes; 4) abusive use damaging the landlord and other tenants; 5) subletting of dwelling against the will of the owner; or 6) owner undertakes construction works to improve or extend the house, whose value $\ge$10% of the fiscal value of the real estate (*valor asignado al inmueble para el pago de la contribución directa*).	
1921-09-15 / 1921-09-19 — Ley 11.157 Locación urbana: Congelación de alquileres; suspensión de desalojos en Capital Federal y en territorios nacionales	Regions: Federal Capital and National Territories. Objects: house, room, or apartment let or sublet.	Setting: during the next 2 years, the rents are fixed at the Jan. 1, 1920 level.		
1923-10-01 / 1923-10-04 — Ley 11.231 Locación Urbana: Prórroga de los contratos hasta el 30/09/24	Objects: house, room, or apartment let or sublet.		Automatic prolongation until Sep. 30, 1924.	
1924-12-02 / 1924-12-10 — Ley 11.318 Locación urbana: prórroga de la ley 11231 hasta el 30 de setiembre de 1925	Validity: prolongation of law 11231 until Sep. 30, 1925.			
1943-06-29 / 1943-07-10 — Decreto 1580/43 Establece rebaja de alquileres a partir del 1/7/43 para la Capital Federal y partidos circunvecinos de la Provincia de Buenos Aires	Regions: whole country. Period: until Dec. 31, 1945. Objects: all contracts (written or verbal), including sublettings and sesions with and without furniture.	Setting: starting on July 1, 1943, rents are fixed at the Dec. 31, 1942 level minus reductions. If no rent at that date is available, the latest rent level is taken. Rents for dwellings built or transformed after Dec. 31, 1942 are set by the arbitration councils. Reductions: 1) 5–20% depending on the rent level, in the Federal Capital and specifically delimitated regions; 2) to be set by the regional authorities in the rest of the country.	Automatic prolongation for 1.5 years. Prohibition to refuse letting of dwellings to families with small children (*hijos menores*). Prohibition to suppress complementary services: heating, lift, central hot water, etc.	
1943-08-20 / 1943-09-16 — Decreto 5893 de 1943 Amplía las disposiciones del decreto núm. 1580 (1), en lo que se refiere a los contratos de locación y sublocación de piezas	Objects: (sub)letting of rooms (piezas). Validity: until Dec. 31, 1945.		Automatic prolongation for 1.5 years. Non-prolongation reasons: 1) non-payment during 3 consecutive periods; or 2) reasons indicated in the Civil Code.	
1944-06-15 / 1944-06-24 — Decreto 15.516 de junio 15 de 1944 (A. de M.) fija normas sobre causas que motivan desalojos de inmuebles sujetos a locación o sublocación or mejoras o reparaciones durante la vigencia del decreto núm. 1580/43	Validity: during validity of the decreto 1580/43.		Eviction due to improvement works is prohibited, unless the house is completely demolished and a new house with a higher rental capacity (*capacidad locativa*) is built.	
1944-10-17 / 1944-10-27 — Decreto 27.736 de octubre 17 de 1944 (A. de M.) declara que los convenios sobre precios y plazos de locación durante la vigencia del decreto núm. 1580 (4), siempre que se ajusten a las disposiciones del Código civil son válidos y no contravienen las prescripciones del art. 12 de dicho decreto		Setting: rent is fixed at the Dec. 1942 level.	Tenant can interrupt rental contract at any time.	
1944-12-06 / 1944-12-12 — Decreto 33.059 de 1944 Sustiye el art. 4 del decreto núm. 1580/43 (1), referente al régimen de alquileres			Automatic prolongation until Dec. 31, 1945. Termination reasons: as in art. 1507 of the Civil Code.	
1945-11-21 / 1945-11-23 — Decreto 29.716 de 21 de noviembre de 1945 (A. de M.) prorroga hasta el 31 de diciembre de 1946 el régimen establecido por los decretos núms. 1580/43 y 33059/44 sobre locación de fincas urbanas		Rent remains unchanged at the level fixed under the decreto 1580/43.	Prolongation of contracts until Dec. 31, 1946.	
1946-09-20 / 1946-09-21 — Ley 12.847 Suspende el trámite de los juicios pendientes de desalojo	Objects: house, room, or apartment with monthly rent $\le$200 pesos. Region: capital of the Republic.		Suspension of eviction judgements for 90 days. Exceptions: municipal orders based on sanitary and security questions.	
1946-09-28 / 1946-10-04 — Ley 12.862 hace extensiva a todo el territorio de la República las disposiciones de la ley núm. 12847 sobre suspensión de desalojos	Region: whole country.			
1946-11-29 / 1946-11-29 — Ley 12886 prorroga las locaciones	Objects: houses, establishments (*locales*), apartments, rooms, and other urban residential premises (*habitaciones urbanas*); urban estates (*fincas urbanas*) subject to legal regime of the Banco Hipotecario Nacional.		Automatic prolongation until Dec. 31, 1947.	
1946-12-22 / 1947-01-03 — Ley 12.926 prorroga las disposiciones de las leyes núms. 12.847 y 12.862 sobre suspensión de desalojos			Extension of the suspension of evictions until June 30, 1947. Exceptions: 1) non-payment; 2) abusive use (*uso abusivo*); or 3) scandals, etc.	
1947-06-27 / 1947-07-02 — Ley 12.991 prorroga hasta el 31 de julio la vigencia de la ley sobre desalojos	Validity: prolongation of the law 12.926 until July 31, 1947.		Extension of the suspension of evictions until July 31, 1947.	
1947-08-06 / 1947-08-18 — Ley 12.998 suspende desalojos hasta el 30/06/49 y fija excepciones	Regions: the whole territory of the Republic. Period: until June 30, 1949. Objects: houses, apartments, rooms, and other urban dwellings; contracts (written or verbal), including sublettings and sesions with and without furniture.		Automatic prolongation until June 30, 1949. Exceptions: 1) non-payment; 2) abusive use; 3) scandals (*escándalo*); 4) owner needs the dwelling for himself and his family, provided that it is his only dwelling and monthly rent $\le$200 pesos, and fiscal value (*valuación fiscal*) of the dwelling at Dec. 31, 1946 $\le$100,000 pesos; 5) owner plans to rebuild the house, having low housing capacity, in order to create more dwellings (5–20 depending on the reconstruction date) having dining room, 2 bedrooms, bathroom, and kitchen with monthly rent $\le$200 pesos; or 6) new dwellings let after this law enters in action.	
1948-08-19 / 1948-08-25 — Ley 13.228 sustituye el inc. c del artículo 2 de la ley 12998 (desalojos)			Non-prolongation reasons: 1) houses already acquired by employees and workers active or retired using loans of the Banco Hipotecario, Instituto Nacional de Previsión Social, or provincial credits; or 2) the owner needs the dwelling for himself and his family, which is his only property.	
1949-07-28 / 1949-08-02 — Ley 13.538 suspende todos los juicios de desalojo hasta el 30/09/49	Objects: houses, apartments, rooms, and other urban residential premises.		Suspension of all eviction cases (*juicios de desalojo*) until Sep. 30, 1949.	
1949-09-29 / 1949-10-05 — Ley 13.581 Régimen de emergencia de las locaciones urbanas	Objects: houses, apartments, and rooms with and without furniture. Duration: until Sep. 30, 1951.	Setting: basic rent is fixed 1) at the previous rent level; 2) by the arbitration councils, if dwelling was not let prior to the enactment of the law.	Automatic prolongation until Sep. 30, 1951. Non-prolongation reasons: 1) (sub)tenant, who rented dwelling during the temporary absence of landlord (tenant); or 2) rental contract is part of other contract. *Post mortem* transfer: upon the tenant’s death, the rental contract is inherited by the family members of the tenant and persons depending on him. Prohibition to suspend or reduced supplementary services provided by the landlord. Termination reasons: 1) non-payment of rent during 2 periods; 2) tenant abuses the dwelling and commits infractions of the rental contract; 3) property belongs to the state; 4) tenant is absent from the dwelling for $\ge$6 months, unless it is required by his profession, function, or *force majeure*; 5) owner needs the dwelling, which is his only property; 6) owner plans to build a house with a larger capacity; or 7) tenant sublets dwelling without authorization of landlord. Minimum duration: 3 years.	1) obligatory registration of vacant dwellings by landlords and subletting tenants within 15 days; 2) landlords are required to let their dwellings within 30 days; and 3) in Federal Capital and National Territories, the authorities can requisition vacant dwellings.
1952-09-30 / 1952-10-06 — Ley 14.178 modifica la ley 13.581 desalojo			Termination reasons: extension of eviction in case of public property.	
1954-09-29 / 1954-10-13 — Ley 14.356 Locación urbana: modificación y prórroga de la ley 13.581	Validity: prolongation of law 13.581 until Sep. 30, 1955.	Updating: 30% increase of basic rents for dwellings completed (*habilitadas*) prior to Jan. 1, 1950.		
1955-12-30 / 1955-12-30 — Decreto-ley 7588/55 Locación urbana: Modificación y prórroga de la ley 13.581	Objects: houses, apartments, and rooms with and without furniture. Duration: until Aug. 31, 1956.	Setting: basic rent is fixed 1) at the previous rent level; or 2) by the arbitration councils, if dwelling was not let prior to the enactment of the law.	Automatic prolongation until Sep. 30, 1951. Non-prolongation reasons: 1) (sub)tenant, who rented dwelling during the temporary absence of landlord (tenant); or 2) rental contract is part of other contract. *Post mortem* transfer: upon the tenant’s death, the rental contract is inherited by the family members of the tenant and persons depending on him. Prohibition to suspend or reduced supplementary services provided by the landlord. Termination reasons: 1) non-payment of rent during 2 periods; 2) tenant abuses the dwelling and commits infractions of the rental contract; 3) property belongs to the state; 4) tenant is absent from the dwelling for $\ge$6 months, unless it is required by his profession, function, or *force majeure*; 5) owner needs the dwelling, which is his only property; 6) owner plans to build a house with a larger capacity; or 7) tenant sublets dwelling without authorization of landlord. Minimum duration: 3 years.	1) obligatory registration of vacant dwellings by landlords and subletting tenants within 15 days; 2) landlords are required to let their dwellings within 30 days; and 3) in Federal Capital and National Territories, the authorities can requisition vacant dwellings.
1957-02-28 / 1957-03-08 — Decreto-ley 2186/57 Régimen de la locación urbana	Objects: houses, apartments, and rooms with and without furniture. Duration: until Sep. 30, 1958. Exceptions: 1) dwellings built since Mar. 1, 1957; 2) contracts concluded since Mar. 1, 1957; 3) hotels, lodging (*hospedajes*), pensions (*pensiones*), and similar establishments; 4) holiday/tourist accommodation; 5) premises let during the absence of the landlord; 6) dwelling is rented as a part of other contract; or 7) tenant or his spouse have enough resources to acquire or rent a dwelling.	Setting: rent is set at the level of monthly rate of ordinary common expenditure (*cuota mensual de los gastos ordinarios comunes*) of unities sold under the ley 13.512. Updating: no restrictions.	Automatic prolongation until Sep. 30, 1958. Non-prolongation reasons: 1) (sub)tenant, who rented dwelling during the temporary absence of landlord (tenant); or 2) rental contract is part of other contract. *Post mortem* transfer: upon the tenant’s death, the rental contract is inherited by the family members of the tenant and persons depending on him. Prohibition to suspend or reduced supplementary services provided by the landlord. Termination reasons: 1) non-payment of rent during 2 periods; 2) tenant abuses the dwelling and commits infractions of the rental contract; 3) tenant is absent from the dwelling for $\ge$6 months, unless it is required by his profession, function, or *force majeure*; 4) owner needs the dwelling, which is his only property; 5) owner plans to build a house with a larger capacity; or 6) tenant sublets dwelling without authorization of landlord. Minimum duration: 2 years, according to the Civil Code.	1) obligatory registration of vacant dwellings by landlords and subletting tenants within 15 days; 2) landlords are required to let their dwellings within 30 days; and 3) in Federal Capital and National Territories, the authorities can requisition vacant dwellings.
1958-05-29 / 1958-05-30 — Ley 14.438 paralización de juicios de desalojo			Between the promulgation of this act and Sep. 30, 1958, suspension of evictions, except those related to non-payment.	
1958-11-15 / 1958-12-09 — Ley 14.775 prórroga del régimen de locaciones y de la paralización de juicios de desalojo y lanzamientos	Validity: prolongation until June 30, 1959 of decreto-ley 2186/57 (1), decreto-ley 9940/57 (2), leyes 14.438 (3) and 14.442 (4).		Suspension of evictions until June 30, 1959.	
1959-06-30 / 1959-07-15 — Ley 14.809 prórroga del régimen de locaciones y de la paralización de juicios de desalojo y lanzamientos	Validity: prolongation until July 31, 1959 of ley 14.775 (1).		Suspension of evictions until July 31, 1959.	
1959-07-31 / 1959-08-01 — Ley 14.821 Régimen de locaciones urbanas	Objects: (sub)letting of premises or their parts, with or without furniture, aimed at residential, commercial, industrial, or any other legal activity. Exceptions: 1) letting of advertisement premises; 2) space or places designed for keeping vehicles, animals, or other objects; 3) letting of rooms in the establishments, which are completely or in part are used for lodging or hotels and family pensions; 4) letting of premises during vacation seasons or for the purposes of tourism; 5) letting of premises during the temporary absence of the landlord; 6) premises that are not used for legal purposes; 7) occupation resulting from accessory clauses of other contracts; 8) the contracts concluded after Mar. 1, 1957 and in the future, provided that in both cases a new or vacant dwelling is let; and 9) lettings, where the owner or his spouse does not possess enough means to cover the minimum life needs, except the tenant is in identical need.	Setting: rent set in the last contract $\le$7% of rateable value (*valuación fiscal*) + taxes and fees for the rented premises. Updating: annual rent increase is 5%. Validity: between July 1, 1959 and Dec. 31, 1963.	Suspension of evictions until Dec. 31, 1963. *Post mortem* transfer: after the death of tenant, the contract can be inherited by his family members or subtenants. Prohibition to suspend or reduced supplementary services provided by the landlord. Termination reasons: 1) non-payment of rent during 2 periods; 2) tenant abuses the dwelling; 3) tenant is absent from the dwelling for $\ge$6 months, unless it is required by his profession, function, or *force majeure*; 4) owner, who became owner prior to Dec. 31, 1950, needs the dwelling, which is his only property; 5) owner plans to build a house with a triple inhabitable capacity and number of dwellings, provided that existing house is >25 y.o.; or 6) tenant sublets dwelling without authorization of landlord.	
1960-11-17 / 1960-12-06 — Ley 15.775 Régimen de locación urbana	Prolongation of law 14.821 until June 1964.	Setting: rent set in the last contract $\le$7% of rateable value + taxes and fees for the rented premises. Updating: annual rent increase is 5%. Validity: between Jan. 1, 1960 and Dec. 31, 1963.	Suspension of evictions until Dec. 31, 1963. *Post mortem* transfer: after the death of tenant, the contract can be inherited by his family members or subtenants. Prohibition to suspend or reduced supplementary services provided by the landlord. Termination reasons: 1) non-payment of rent during 2 periods; 2) tenant abuses the dwelling; 3) tenant is absent from the dwelling for >6 months, unless it is required by his profession, function, or force majeure; 4) owner, who became owner prior to Dec. 31, 1950, needs the dwelling, which is his only property; 5) owner plans to build a house with a triple inhabitable capacity and number of dwellings, provided that existing house is >30 y.o.; or 6) tenant sublets dwelling without authorization of landlord.	
1964-09-29 / 1964-09-30 — Ley 16.485 Locación urbana; ampliación de la prórroga del régimen de emergencia hasta el 31 de diciembre de 1964	Validity: prolongation until Dec. 31, 1964 of decreto-ley 8058/63.		Suspension of evictions until Dec. 31, 1964.	
1964-12-30 / 1965-01-11 — Ley 16.654 Locación urbana; prórroga del régimen de emergencia hasta el 31 de julio de 1965	Validity: prolongation until July 31, 1965 of law 14.821 modified by the law 15.775.		Suspension of evictions until July 31, 1965.	
1965-10-01 / 1965-10-11 — Ley 16.739 régimen de locación urbana	Objects: (sub)letting of premises, with or without furniture, aimed at residential, commercial, industrial, or any other legal activity. Exceptions: 1) contracts concluded from Mar. 1, 1957 on; 2) contracts, in which the state and its bodies are tenants; or 3) new or enabled (*habilitadas*) dwellings between Jan. 1, 1954 and Feb. 28, 1957.	Setting: basic rent is set at the level of rent indicated in the last contract, but not earlier than 1943. Updating: between Jan. 1, 1966 and Dec. 31, 1970, the cumulative rent increase can be 10%.	Automatic prolongation until Dec. 31, 1970. Non-prolongation reasons: 1) dwelling was let during a temporary absence of the landlord; 2) dwelling is used for illegal purposes; 3) rental contract is part of other contract. Termination reasons: 1) non-payment; 2) tenant abuses the real estate; 3) tenant uses the dwelling for other purposes than stipulated in the contract; 4) tenant is absent in the dwelling for $\ge$6 months without justification; 5) owner needs dwellings for himself, his family, descendants/ascendants; 6) owner plans to rebuild the house; 7) transfer of (sub)let property without authorization of landlord. Death of tenant: contract is inherited by his 1) family members; or 2) subtenants. Prohibition to suspend or reduced supplementary services provided by the landlord.	
1967-07-27 / 1967-08-02 — Ley 17.368 Locación urbana; retorno al sistema del Código Civil en los nuevos contratos	Objects: all rental contracts concluded after the publication of this law, which are not continuation of the anterior ones.	Intertenancy decontrol.		
1970-12-29 / 1971-01-01 — Ley 18.880 Locación urbana - régimen	Objects: all (sub)letting of urban real estates with or without furniture assigned exclusively for residential purposes. Validity: between Jan. 1, 1971 and Dec. 31, 1974. Exceptions: 1) contracts concluded from Mar. 1, 1957 on with persons who are not previous tenants; 2) new dwellings completed since Mar. 1, 1957; 3) dwellings enabled since Jan. 1, 1954; 4) tenant has sufficient economic resources to buy or rent another dwelling, which would be adequate to his needs, even if worse than the current one.	Setting: rent is fixed at 20% of basic rent in 1971 to 80% in 1974. Basic rent = initial rent $\times$ adjustment factor. Initial rent = rent paid in min{first month of renting, Jan. 1, 1943}. Adjustment factor varies from 260 for 1943 to 23 for 1957. Updating: rent growth rate = rate of increase of the official index of living costs (*índice de costo de vida*). Tenant, whose income results from his personal labor, can ask the landlord to readjust the rent s.t. it does not exceed 25% of the tenant’s income. The decision about readjustment is made by judges, who can allow it to be 35%, if the dwelling manifestly exceeds the needs of the tenant and persons cohabiting with him.	Automatic prolongation until Dec. 31, 1974. Termination reasons: 1) non-payment; 2) tenant abuses the real estate; 3) tenant uses the dwelling for other purposes than stipulated in the contract; 4) tenant is absent from the dwelling for $\ge$4 consecutive months without justification or 12 months with justification; 5) owner needs dwelling for himself, his family, descendants/ascendants; 6) owner plans to rebuild the house; or 7) transfer of (sub)let property without authorization of landlord. Death of tenant: contract is inherited by his 1) family members; or 2) subtenants.	
1971-12-31 / 1971-12-31 — Ley 19.405 prórroga de contratos. Reducción de los porcentajes de aumentos. Sustitución de los artículos 2 y 5 de la ley 18880		Setting: rent is fixed at 20% of basic rent in 1971 to 80% in 1975.	Automatic prolongation until Dec. 31, 1975.	
1973-12-21 / 1974-01-21 — Ley 20.625 Locaciones urbanas - Régimen de emergencia	Objects: all (sub)letting of urban real estates with or without furniture contracted prior to enactment of this law. Validity: from Jan. 1, 1974. Exceptions: 1) contracts concluded after Dec. 31, 1973; 2) rental contracts conditioned upon labor contracts; or 3) tenant has sufficient economic resources to buy or rent another dwelling, which would be adequate to his needs.	Setting: rent is fixed at the Dec. 31, 1973 level. Updating: rent growth rate = rate of increase of the official index of wage of industrial workers (*indice salarial del peón industrial*) in the Federal Capital during the last 6 months. Tenant can ask the landlord to readjust the rent s.t. it does not exceed 20% of the tenant’s income. Rent has to be expressed in domestic currency, not in foreign currencies or gold. Obligatory insurance of rent (*seguro de garantía de alquileres*) with the policy paid in equal parts by the tenant and landlord.	Automatic prolongation until Dec. 31, 1975. Termination reasons: 1) non-payment during 2 months; 2) tenant abuses the real estate, uses it for illegal, dishonest, contradicting the good customs, violates the normal cohabitation; 3) tenant uses the dwelling for other purposes than stipulated in the contract; 4) tenant is absent from the dwelling for >4 consecutive months without justification or 12 months with justification; 5) owner needs dwelling for himself, his family, descendants/ascendants; 6) owner plans to rebuild the house, provided that the living space or the number of housing units (*unidades funcionales de vivienda*) in the new building will be 3 times larger than in the old one; or 7) transfer of (sub)let property without authorization of landlord. Death of tenant: contract is inherited by his 1) family members; or 2) subtenants.	
1976-06-29 / 1976-07-01 — Ley 21.342 Normalización de locaciones urbanas. Régimen que reemplaza al instituído por la Ley 20.625 y sus prórrogas	Objects: all (sub)letting of urban real estates with or without furniture contracted prior Jan. 1, 1974. Validity: gradual liberation until Nov. 30, 1979 of the contracts concluded prior to Jan. 1, 1979, first the latest (between Aug. 3, 1967 and Dec. 31, 1973) contracts are liberated, then the earliest ones (prior to Mar. 1, 1957). Exceptions: 1) rental contracts conditioned upon other contracts; 2) tenant has sufficient economic resources to buy or rent another dwelling, which would be adequate to his needs, even if worse than the current one; or 3) contracts concluded since Jan. 1, 1974.	Setting: 1) freely, if contract concluded since Jan. 1, 1974; 2) rent is fixed at 12.5–100% of rental value (*valor locativo*) depending on the date, when contract was concluded. Rental value = initial rent $\times$ adjustment factor. Initial rent = rent paid in min{first month of renting, Jan. 1, 1943}. Updating: rent growth rate = rate of increase of the official index of wage of industrial workers in the Federal Capital during the preceding quarter. Rent increases can be made every quarter. Tenant can ask the landlord to readjust the rent s.t. it does not exceed 25% of the tenant’s income. Rent must cover all the expenses of the landlord and provide him net revenue = either 50% of these expenses or 10% of the rental value. Landlord can either require a guarantee payment from the tenant or insurance of rent, in which case the policy is paid in equal parts by the tenant and landlord.	Termination reasons: 1) non-payment during 2 periods; 2) tenant is absent from the dwelling for >4 consecutive months without justification or 12 months with justification; 3) owner needs dwelling for himself, his family, descendants/ascendants; 4) owner plans to rebuild the house, provided that the number of housing units in the new building will be 3 times bigger than in the old one (each unit must have separate entrance, at least 1 room, cooking space, and bathroom). *Post mortem* transfer: contract is inherited by his 1) family members; or 2) subtenants.	
/ 1979-12-01 — Ley 21.342	End of liberalization.			
1984-09-20 / 1984-12-16 — Ley 23.091 Locaciones urbanas	Objects: urban lettings.	Setting: not specified. Rent can be expressed in foreign currency (*moneda de curso legal*). Updating: rent growth rate = growth rate of an official price index. Guarantee: can be expressed in foreign currency.	Termination reasons: non-payment of rent during 2 periods. Minimum contract duration: 2 years for dwellings with or without furniture. Exception: holiday dwellings with contract duration up to 6 months. Death of tenant: contract is inherited by persons who cohabited with the tenant.	Municipality of Buenos Aires and national territories are empowered to establish differential treatment (*gravámenes diferenciales*) wrt vacant dwellings.
1987-11-12 / 1987-12-03 — Ley 23.542 Locaciones urbanas: Reducción porcentual	Object: urban lettings.	Updating: in Oct. 1987, rent growth rate = growth rate of a price index minus 10 percentage points. This change affects the rent until the end of the corresponding contract.		
1989-09-29 / 1989-10-06 — Ley 23.747 Disposiciones para los locadores y locatarios comprendidos en la Ley N$^{\circ }$ 23.091 y en las normas del régimen general aplicable a las locaciones urbanas cualquiera fuere su destino	Object: urban lettings.	Setting: rent in Oct. 1989 = rent in Sep. 1989. If landlord disagrees with resulting rent level, he can apply to court, which can set new rent based on the expert estimates of the real rental value of the dwelling. Updating: consecutive non-monthly rent adjustments shall not account for rent growth rate in Oct. 1989.		
1991-03-27 / 1991-04-01 — Ley 23.928 Convertibilidad del Austral	Validity: from Apr. 1, 1991 on.	Updating: rent indexation is prohibited. Rents agreed upon in the contracts preceding Apr. 1, 1991 can be increased by, at most, change in the exchange rate of Austral wrt US dollar between max{date of contract, May 1990) and Apr. 1, 1991 + 12%.		
2002-01-06 / 2002-01-06 — Ley 25.561 Emergencia pública y reforma del sistema cambiario		Setting: payment obligations that existed on Jan. 6, 2002 and expressed in US dollars or other foreign currencies are converted to domestic currency with exchange rate 1 US dollar = 1 peso. Updating: rent indexation is prohibited. National govt is empowered to regulate prices of the critically important goods and services.		
2002-02-03 / 2002-02-04 — Decreto 214/02 Decreto de Necesidad y Urgencia del Poder Ejecutivo Nacional (“Reordenamiento del Sistema Financiero. Nuevas medidas económicas. Pesificación”)		All monetary obligations expressed in US dollars or other foreign currency are to be converted using an exchange rate 1 US dollar = 1 peso.		
2014-12-01 / 2015-08-01 — Ley 26.994 Código Civil y Comercial de la Nación		Setting: rent is set either by negotiation of the contract participant or by a third party (e.g., judge). Updating: rent can be pegged to a value of some foreign currency or commodity (e.g., value of the US dollar or other foreign currency, the price of the NAFTA, the price of bricks or cement).	Minimum duration: 2 years. Maximum duration: 20 years. Death of tenant: contract is inherited by persons who cohabited with the tenant.	
2020-03-29 — DECNU-2020-320-APN-PTE — Alquileres	Objects: lease contracts for 1) properties intended for single urban or rural dwelling; 2) rooms for family or personal housing in pensions, hotels, or other similar accommodation; 3) buildings intended for cultural or community activities; 4) rural buildings destined for small family productions and small agricultural productions; 5) real estate rented by people adhered to the Monotributo regime, destined for the provision of services, commerce, or industry; 6) buildings rented by self-employed professionals for the exercise of their profession; 7) properties rented by Micro, Small and Medium-sized Enterprises, intended for the provision of services, commerce, or industry; 8) properties rented by Worker Cooperatives or Recovered Companies registered in the *Instituto nacional de asociativismo y economía social*. Validity: until Sep. 30, 2020.	Setting: the rent freeze at the March 2020 level.	Suspend the execution of court judgments whose purpose is the eviction of real estate, provided that the litigation results from the breach of the obligation to pay in a lease contract. Automatic prolongation: Extend the validity of the contracts for the location of the properties, which expired after March 20, 2020 and for contracts whose expiration is scheduled before September 30, 2020	
2020-06-11 — Ley 27551 — Modificación Código Civil y Comercial de la Nación	Objects: residential leases.	Updating: rent increases are pegged to an average of the growth rates of the consumer price index and wage index of stable workers (*remuneración imponible promedio de los trabajadores estables*)	Minimum duration: 3 years

**Table 5:** Results of augmented Dickey-Fuller stationarity tests

Variable	No drift no trend	With drift no trend	With drift and trend
*Levels*			
LRRent	0.237	0.691	0.775
RIRate	0.010	0.060	0.195
LPop	0.979	0.010	0.875
Lcgdppc	0.984	0.777	0.020
LPermits	0.087	0.798	0.010
*Differences*			
DLRRent	0.010	0.010	0.024
DRIRate	0.010	0.010	0.010
DLPop	0.282	0.413	0.010
DLcgdppc	0.010	0.010	0.010
DLPermits	0.010	0.010	0.010

**Table 6:** Estimation results of OLS and MARS models for real rent growth

Variable	Linear model		MARS	
	(1)	(2)	(3)	(4)
Constant	0.089	0.270	−0.035$$^{***}$$	−0.020$$^{***}$$
	(0.069)	(0.271)	(0.000)	(0.000)
DLRRent_L1	− 0.127	− 0.147		
	(0.143)	(0.145)		
RIRate_L1	−0.003$$^{*}$$	−0.003$$^{*}$$	−0.003$$^{***}$$	−0.003$$^{***}$$
	(0.002)	(0.002)	(0.000)	(0.000)
DDLPop_L1	12.591	13.568		
	(20.511)	(21.268)		
DLgdppc_L1	0.397	0.417		
	(0.595)	(0.619)		
DLPermits_L1	− 0.060	− 0.059		
	(0.093)	(0.097)		
*D*_meth1	− 0.019	− 0.085		
	(0.084)	(0.121)		
*D*_meth2	− 0.142$$^{**}$$	−0.200$$^{*}$$		−0.103$$^{***}$$
	(0.065)	(0.117)		(0.000)
*D*_meth3	− 0.068	− 0.073		
	(0.124)	(0.118)		
*D*_meth4	− 0.076	− 0.146		
	(0.072)	(0.147)		
*D*_meth5	0.077	0.070		
	(0.075)			
*D*_meth6	− 0.065	− 0.071		
RMRI_L1	− 0.172			
	(0.168)			
Rent_laws_L1		0.028		
		(0.223)		
Tenure_security_L1		− 0.530		
		(0.703)		
Rationing_L1	− 0.061	0.898		
	(0.509)	(1.650)		
‘h(RMRI_L1-0.541667)‘			− 0.464$$^{***}$$	
			(0.000)	
Observations	90	90	90	90
Adjusted ${R}^{2}$	0.049	0.048	0.104	0.101

$$^{*}{p}<0.1$$; $$^{**} {p}<0.05$$; $$^{***} {p}<0.01$$

**Table 7:** Estimation results of OLS model for real rent growth with different lags of regulation indices

	Dependent variable: DLRRent					
	(1)	(2)	(3)	(4)	(5)	(6)
Constant	0.131	− 0.025	0.154	− 0.327	0.123	− 0.210
	(0.128)	(0.285)	(0.144)	(0.247)	(0.145)	(0.296)
DLRRent_L1	− 0.235$$^{**}$$	− 0.220$$^{**}$$	− 0.258$$^{***}$$	− 0.263$$^{**}$$	− 0.255$$^{**}$$	− 0.270$$^{**}$$
	(0.095)	(0.093)	(0.093)	(0.118)	(0.102)	(0.137)
RIRate_L2	− 0.003	− 0.002	− 0.004	− 0.001	− 0.004	− 0.002
	(0.004)	(0.003)	(0.004)	(0.003)	(0.004)	(0.003)
DLPop_L2	− 10.455	− 8.580	− 7.542	− 5.720	− 10.284	− 9.976
	(10.645)	(10.157)	(11.224)	(12.359)	(12.962)	(13.546)
DLgdppc_L2	0.397	0.421	0.517	0.448	0.458	0.530
	(0.544)	(0.534)	(0.571)	(0.554)	(0.558)	(0.562)
DLPermits_L2	− 0.070	− 0.068	− 0.105	− 0.039	− 0.074	− 0.057
	(0.081)	(0.080)	(0.098)	(0.074)	(0.085)	(0.075)
DLCPI_L2	0.017	0.031	0.006	0.068	0.007	0.049
	(0.111)	(0.102)	(0.114)	(0.101)	(0.111)	(0.088)
Left_right_BLS_L2	− 0.002	− 0.002	0.0002	0.005	0.015	0.012
	(0.055)	(0.057)	(0.046)	(0.049)	(0.041)	(0.044)
*D*_meth1	0.196	0.214	0.119	0.248	0.211	0.325
	(0.189)	(0.200)	(0.207)	(0.234)	(0.221)	(0.269)
*D*_meth2	− 0.0003	0.033	− 0.036	0.117	− 0.012	0.118
	(0.103)	(0.123)	(0.108)	(0.137)	(0.111)	(0.168)
*D*_meth3	0.062	0.056	0.033	0.044	0.054	0.079
	(0.144)	(0.149)	(0.142)	(0.138)	(0.124)	(0.128)
*D*_meth4	0.024	0.070	− 0.024	0.150	0.007	0.156
	(0.132)	(0.170)	(0.145)	(0.192)	(0.144)	(0.222)
*D*_meth5	0.176	0.169	0.161	0.146	0.151	0.149
	(0.107)	(0.111)	(0.098)	(0.097)	(0.099)	(0.099)
RMRI_L2	− 0.184					
	(0.251)					
Rent_laws_L2		− 0.184				
		(0.134)				
Tenure_security_L2		0.243				
		(0.636)				
Rationing_L2	− 0.090	− 0.797				
	(0.509)	(1.440)				
RMRI_L3			−0.284			
			(0.237)			
Rent_laws_L3				− 0.445$$^{**}$$		
				(0.203)		
Tenure_security_L3				0.968$^{*}$		
				(0.535)		
Rationing_L3			− 0.087	− 2.346$^{*}$		
			(0.703)	(1.360)		
RMRI_L4					− 0.179	
					(0.227)	
Rent_laws_L4						− 0.326
						(0.263)
Tenure_security_L4						0.689
						(0.705)
Rationing_L4					0.012	− 1.520
					(0.625)	(1.522)
Observations	86	86	86	86	86	86
${R}^{2}$	0.193	0.198	0.220	0.261	0.193	0.208
Adjusted ${R}^{2}$	0.034	0.026	0.067	0.102	0.034	0.038

$$^{*}{p}<$$0.1; $$^{**} {p}<0.05$$; $$^{***} {p}<0.01$$

**Table 8:** Estimation results of OLS model for real rent growth with different proxies for economic growth and demographics

	Dependent variable: DLRRent					
	(1)	(2)	(3)	(4)	(5)	(6)
Constant	0.131	−0.025	0.130	− 0.030	0.036	− 0.156
	(0.128)	(0.285)	(0.126)	(0.289)	(0.081)	(0.256)
DLRRent_L1	− 0.235$$^{**}$$	− 0.220$$^{**}$$	− 0.237$$^{**}$$	− 0.221$$^{**}$$	− 0.225$$^{**}$$	− 0.203$$^{**}$$
	(0.095)	(0.093)	(0.095)	(0.093)	(0.095)	(0.088)
RIRate_L2	− 0.003	− 0.002	− 0.003	− 0.002	− 0.003	− 0.002
	(0.004)	(0.003)	(0.004)	(0.003)	(0.003)	(0.003)
DLPop_L2	− 10.455	− 8.580	− 10.887	− 8.983		
	(10.645)	(10.157)	(10.579)	(10.063)		
DLgdppc_L2	0.397	0.421				
	(0.544)	(0.534)				
DLMarriage_CF_L2					0.437	0.467
					(0.338)	(0.319)
DLRGDP_own_L2			0.468	0.498	0.368	0.408
			(0.540)	(0.532)	(0.502)	(0.486)
DLPermits_L2	− 0.070	− 0.068	− 0.075	− 0.074	− 0.063	− 0.060
	(0.081)	(0.080)	(0.081)	(0.080)	(0.077)	(0.076)
DLCPI_L2	0.017	0.031	0.021	0.037	0.013	0.032
	(0.111)	(0.102)	(0.109)	(0.100)	(0.107)	(0.098)
Left_right_BLS_L2	− 0.002	− 0.002	− 0.001	− 0.001	0.010	0.011
	(0.055)	(0.057)	(0.055)	(0.057)	(0.053)	(0.055)
*D*_meth1	0.196	0.214	0.197	0.215	0.018	0.084
	(0.189)	(0.200)	(0.190)	(0.201)	(0.057)	(0.103)
*D*_meth2	− 0.0003	0.033	− 0.002	0.032	− 0.106	− 0.042
	(0.103)	(0.123)	(0.103)	(0.124)	(0.080)	(0.104)
*D*_meth3	0.062	0.056	0.061	0.055	− 0.018	− 0.013
	(0.144)	(0.149)	(0.143)	(0.148)	(0.151)	(0.151)
*D*_meth4	0.024	0.070	0.019	0.066	−0.047	0.031
	(0.132)	(0.170)	(0.132)	(0.170)	(0.096)	(0.151)
*D*_meth5	0.176	0.169	0.172	0.166	0.119	0.116
	(0.107)	(0.111)	(0.106)	(0.110)	(0.096)	(0.101)
RMRI_L2	− 0.184		− 0.184		− 0.186	
	(0.251)		(0.251)		(0.239)	
Rent_laws_L2		− 0.184		− 0.187		− 0.212
		(0.134)		(0.136)		(0.146)
Tenure_security_L2		0.243		0.253		0.369
		(0.636)		(0.643)		(0.607)
Rationing_L2	− 0.090	− 0.797	− 0.084	− 0.812	− 0.083	− 1.043
	(0.509)	(1.440)	(0.510)	(1.452)	(0.521)	(1.398)
Observations	86	86	86	86	86	86
${R}^{2}$	0.193	0.198	0.195	0.200	0.202	0.211
Adjusted ${R}^{2}$	0.034	0.026	0.037	0.029	0.045	0.041

$$^{*} {p}<0.1$$; $$^{**} {p}<0.05$$; $$^{***} {p}<0.01$$

## Footnotes

1 Ley de alquileres y reforma del CC y CN; http://www.senado.gov.ar/parlamentario/parlamentaria/374144/downloadPdf.

2 Santiago Filipuzzi (25th of November 2021) “Sergio Massa dijo que la ley de alquileres “fracasó” y llamó a discutir un nuevo proyecto en el Congreso” La Nación.

3 These tests are carried out using the **R** packages *sandwich* and *car*, respectively.

4 The test is conducted using the **R** package *lmtest*.

5 The normality test is carried out using the **R** package *shapiro.test*.

6 These are estimated using the *lm.boot* function of the **R** package *simpleboot*.

7 As seen in our literature review in Table 1, rent control can lead to all sorts of undesirable effects. In addition, Kholodilin and Kohl (2021) show that rental regulations lead to higher homeownership rates in the long run, thus destroying the rental housing market.
